# Re: Interpreting epidemiologic studies of colorectal cancer prevention

**DOI:** 10.1007/s10654-023-01065-6

**Published:** 2023-11-08

**Authors:** Hermann Brenner, Michael Hoffmeister

**Affiliations:** Division of Clinical Epidemiology and Aging Research, German Cancer Research Center (DKFZ), Im Neuenheimer Feld 581, 69120 Heidelberg, Germany

In a recent analysis [1], we illustrated substantial underestimation of the effects of screening colonoscopy in reducing colorectal cancer (CRC) incidence by the inclusion of non-preventable prevalent cases in the outcome measure of CRC incidence in the NordICC trial, the first and so far only randomized trial on this topic [2]. In a comment on this analysis [3], Song and Bretthauer acknowledge that prevalent cases are an issue, but nevertheless conclude that „prevalent cancers at screening should be counted in clinical trials because there are no reliable statistical analyses which can tease out the true screening benefits without counting them“. However, as shown in our analysis, even if the exact numbers of prevalent cases cannot be determined with certainty among trial participants not undergoing screening colonoscopy, the current practice of counting prevalent cases in clinical trials inevitably leads to substantial underestimation of reported screening effects in all theoretically possible and plausible scenarios. This underestimation most likely explains most of the apparent discrepancy between the tremendous reduction of CRC incidence that has exclusively been observed in the screening age population in the US [4] and the reported small magnitude of screening effects from the NordICC trial. Hence, even if one refrains from correcting for „prevalence bias“ due to uncertainties about its exact magnitude in the reporting of trial results, at the very least the likely very substantial underestimation of screening effects on reducing risk of CRC needs to be acknowledged in the interpretation of the trial results. Otherwise reported trial results will misinform rather than inform stakeholders in the healthcare system, researchers, clinicians and people interested in cancer prevention on the magnitude of screening effects and unduly undermine the large potential of CRC screening in reducing the severe burden of one of the most common cancers globally.
